# Transformation of acute kidney injury to chronic kidney disease: the interaction between mitophagy and NLRP3 inflammasome

**DOI:** 10.3389/fmolb.2025.1643829

**Published:** 2025-09-24

**Authors:** Yixin Zhu, Chenxi Lv, Yanheng Qiao, Hanqi Yang, Wentong Lin, Xuchen Wang, Yueqi Zhang, Bo Yang

**Affiliations:** ^1^ First Teaching Hospital of Tianjin University of Traditional Chinese Medicine, Tianjin, China; ^2^ National Clinical Research Center for Chinese Medicine Acupuncture and Moxibustion, Tianjin, China

**Keywords:** AKI, CKD, mitophagy, NLRP3 inflammasome, targeted drugs

## Abstract

Acute kidney injury (AKI) and chronic kidney disease (CKD) are closely interrelated renal disorders, where AKI frequently progresses to CKD, resulting in irreversible loss of renal function. In recent years, the roles of the NLRP3 inflammasome and mitophagy in the AKI-to-CKD transition have attracted significant attention. As a crucial component of the innate immune system, the NLRP3 inflammasome promotes AKI-to-CKD progression by mediating inflammatory responses and cellular pyroptosis during renal injury. Conversely, mitophagy exerts renoprotective effects through the selective removal of damaged mitochondria, maintenance of cellular homeostasis, and alleviation of inflammation and oxidative stress. Studies demonstrate that NLRP3 activation is closely associated with mitochondrial dysfunction, while mitophagy can suppress NLRP3 activation by clearing damaged mitochondria, establishing a negative feedback regulatory mechanism. During the AKI phase, mitochondrial damage and excessive NLRP3 activation exacerbate renal tubular epithelial cell injury and inflammatory responses. Concurrently, persistent NLRP3 activation and impaired mitophagy lead to chronic inflammation and fibrosis, accelerating the transition from AKI to CKD. Therefore, targeting the NLRP3 inflammasome and modulating mitophagy may emerge as novel therapeutic strategies for AKI-to-CKD transition. This review focuses on elucidating the molecular mechanisms between mitophagy and the NLRP3 inflammasome, along with related targeted therapies, to provide new insights for preventing AKI progression to CKD.

## 1 Introduction

Acute kidney injury (AKI) is a clinical syndrome characterized by a sudden decline or loss of kidney function (Turgut et al., 2023). Its global average mortality rate reaches 23% (Kellum et al., 2021), while ICU patients face an even higher hospital mortality rate of 43.18% (Havaldar et al., 2024). Epidemiological surveys indicate that approximately 850 million people worldwide suffer from various kidney diseases (Francis et al., 2024). According to the Global Burden of Disease Study, chronic kidney disease (CKD) related mortality rose from 15.95 to 18.35 per 100,000 people between 1990 and 2019 (Shahbazi et al., 2024), reflecting a growing disease burden.

Both AKI and CKD are pathophysiologically interconnected syndromes, often leading to complications such as cardiovascular disease, end-stage renal disease, reduced quality of life, and disability (Chawla et al., 2014). Recent studies indicate that CKD develops in AKI patients at a rate of 25.8 cases per 100 person-years (James et al., 2019). Notably, 24.6% of AKI patients progress to CKD within 3 years, demonstrating that incomplete AKI recovery significantly impacts long-term prognosis (Horne et al., 2017). The AKI-to-CKD transition involves multiple pathological mechanisms, including sustained inflammation, chronic hypoxia, and maladaptive tubular repair processes (Yeh et al., 2024). Consequently, developing targeted interventions to disrupt this progression, based on underlying molecular pathways, has emerged as a critical focus in nephrology research.

Inflammatory responses and mitochondrial dysfunction represent key mechanisms driving the AKI-to-CKD transition (Chang et al., 2024; Yeh et al., 2024). Mitochondrial dysfunction promotes oxidative stress, cellular apoptosis, and inflammatory cascade amplification, while persistent inflammation accelerates renal fibrosis, establishing a pathological “injury-inflammation-fibrosis” cycle (Chang et al., 2024). Recent studies in renal toxicity and ischemia-reperfusion rat models have elucidated the protective role of mitophagy (Fontecha-Barriuso et al., 2022). As a crucial quality control mechanism, mitophagy reduces reactive oxygen species (ROS) production by eliminating damaged organelles and significantly suppresses NLRP3 (NOD-like receptor family pyrin domain containing 3) inflammasome activation (Fontecha-Barriuso et al., 2022). The activated NLRP3 inflammasome conversely worsens mitochondrial dysfunction and stimulates additional ROS generation, thereby creating a self-perpetuating “mitochondrial damage-inflammation activation” loop (Lin et al., 2021). This mechanism suggests that targeted regulation of the mitophagy-NLRP3 inflammasome axis could represent a novel therapeutic strategy to disrupt the pathological cycle. Such an approach may provide new directions for preventing and treating the AKI-to CKD-transition.

This review systematically examines the molecular mechanisms underlying the mitochondrial-inflammatory circuit in the AKI-to-CKD transition. By evaluating its translational medical value as a therapeutic target, we aim to establish a theoretical foundation for developing innovative renal protection strategies.

## 2 The pathophysiology of AKI-to-CKD transition

Animal models of ischemia-reperfusion injury (IRI) (Le Clef et al., 2016), nephrotoxic drug exposure (Katagiri et al., 2016), and contrast agent induction (Tsogbadrakh et al., 2025) effectively mimic the pathological progression from AKI to CKD. Post-AKI renal repair outcomes depend on two critical factors: the severity of initial injury and the adaptability of subsequent repair mechanisms. Mild injury typically activates adaptive repair mechanisms, enabling full renal functional recovery. However, severe injury often leads to maladaptive repair. This process involves interconnected pathological cascades, including tubular epithelial cell (TECs) damage and dysfunction, microvascular injury, and endothelial dysfunction, which constitute the core pathological basis for AKI-to-CKD transition (Kurzhagen et al., 2020).

### 2.1 TEC injury and dysfunction

Proximal tubule S3 segment TECs are particularly sensitive to ischemic and nephrotoxic injury due to their high metabolic rate and oxygen demand (Funk and Schnellmann, 2012). These cells are key drivers of the AKI-to-CKD transition (Liu et al., 2018). Injured TECs release damage-associated molecular patterns (DAMPs) and pathogen-associated molecular patterns (PAMPs). These molecules activate the NF-κB pathway via Toll-like receptors (TLRs), thereby inducing NLRP3 inflammasome activation. This leads to the secretion of large amounts of pro-inflammatory factors (such as IL-1β, IL-18, TNF-α) and chemokines (such as CCL2, CCL5) (Liu et al., 2018), initiating an inflammatory cascade, and recruiting and activating immune cells such as neutrophils, macrophages, and dendritic cells (Kim et al., 2019; Lee et al., 2024). These immune cells, particularly activated macrophages, further release pro-inflammatory and pro-fibrotic factors (such as TGF-β, IL-6). These factors exacerbate tissue damage and promote the fibrotic process (Lee et al., 2017; Komada and Muruve, 2019; Meng et al., 2025).

Injured TECs undergo dedifferentiation, manifested by the loss of epithelial markers and the acquisition of mesenchymal markers (such as α-SMA). These cells exhibit partial epithelial-mesenchymal transition (EMT) features (Chang-Panesso and Humphreys, 2017). They acquire migratory and pro-fibrotic capabilities, promoting excessive deposition of the extracellular matrix (ECM) (Kurzhagen et al., 2020; Xu et al., 2025). Persistent inflammation and EMT collectively contribute to progressive tubular atrophy and interstitial fibrosis (Ferenbach and Bonventre, 2015).

### 2.2 Microvascular injury and endothelial dysfunction

Renal injury leads to microcirculatory dysfunction, causing capillary rarefaction, endothelial cell damage, and pericyte detachment (Jiang et al., 2020). Renal capillary endothelial cells may undergo Endothelial-to-Mesenchymal Transition (EndMT), further compromising vascular integrity and exacerbating tissue hypoxia and tubular injury (Lovisa et al., 2020). The resulting chronic hypoxic state persistently stimulates pro-fibrotic signaling pathways, serving as a key microenvironmental factor in CKD progression. The inflammatory response initiated by injured TECs persists and amplifies, while infiltrating immune cells (particularly macrophages) polarize into pro-inflammatory/pro-fibrotic phenotypes within the injured microenvironment, driving excessive ECM deposition (Lee et al., 2017; Lee et al., 2024). These processes ultimately converge into progressive renal interstitial fibrosis.

Although fibrosis may exert a protective effect by encapsulating irreversibly damaged areas and confining injury spread (Kaissling et al., 2013; Guzzi et al., 2019), extensive and persistent fibrosis disrupts normal renal parenchymal architecture. This disruption leads to nephron loss and glomerulosclerosis, constituting the hallmark pathological alteration of CKD (Chawla et al., 2014; Kurzhagen et al., 2020).

Recent studies emphasize that a vicious cycle exists between mitochondrial dysfunction (e.g., accumulated damaged mitochondria and excessive ROS production) and hyperactivated NLRP3 inflammasomes in injured TECs (Yang et al., 2024; Wu M. et al., 2025). This cycle serves as a key molecular mechanism driving maladaptive repair and the AKI-to-CKD transition. Maintaining the balance between mitochondrial homeostasis (such as clearing damaged mitochondria via efficient mitophagy) and suppression of NLRP3 inflammasome overactivation is crucial for promoting adaptive repair and blocking progression to CKD.

## 3 Mitophagy and the NLRP3 inflammasome in the kidney

**FIGURE 1:**
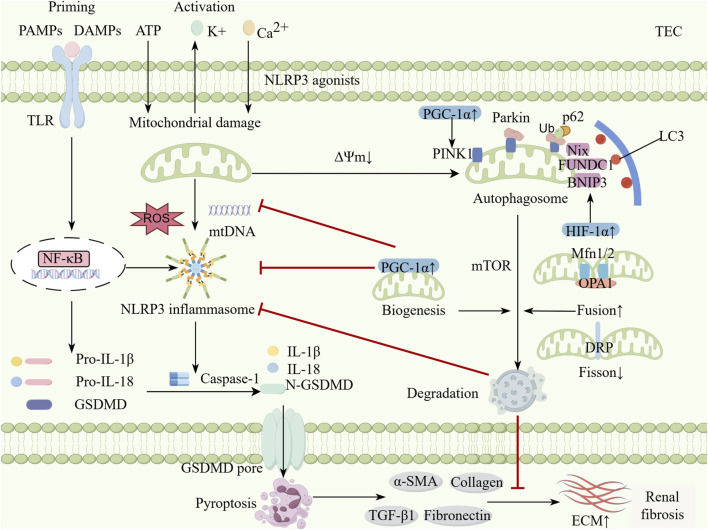
The interplay between mitophagy and NLRP3 inflammasome in kidney diseases. Classic NLRP3 inflammasome activation is a two-step process. During the priming phase, TLR can recognize DAMPs and PAMPs produced by continuous exposure to kidney disease and activate the NF-κB pathway to upregulate the expression of NLRP3, pro-IL-1β and pro-IL-18. During the activation phase, multiple stimulis trigger the assembly of inflammasomes to activate Caspase-1 by inducing K^+^ efflux, Ca^2+^ influx, mitochondrial dysfunction, ROS and mtDNA release. Activated Caspase-1 cleaves pro-IL-1β and pro-IL-18 into activated forms and cracks GSDMD, whose N-terminal fragments form pores in the cell membrane and initiate pyroptosis. Moderate mitophagy can help inhibit excessive inflammation and maintain renal tubular epithelial cell homeostasis. This process mainly relies on the PINK1-Parkin pathway, receptor-mediated pathways (such as BNIP3, NIX, and FUNDC1) and the dual regulation of mTOR. Moderate mitochondrial dynamics and mitochondrial biogenesis can also inhibits the activation of the NLRP3 inflammasome by modulating mitophagy. The activation of mitophagy inhibits the NLRP3 inflammasome and reduces pyroptosis, which decreases the profibrotic signal (α-SMA, Coll-1, FN1, TGFβ-1) and delaying the progression of renal fibrosis (By Figdraw). TEC, Tublular epithelial cell; PAMPs, Pathogen-associated molecular patterns; DAMPs, Danger-associated molecular patterns; TLR, Toll-like receptor; NF-κB, Nuclear factor-kappaB; ROS, Reactive oxygen species; NLRP3, Nucleotide-binding oligomerization domain-like receptor protein 3; IL, Interleukin; GSDMD, Gasdermin D; N-GSDMD, N-terminal Gasdermin D; PINK, PTEN-induced putative kinase 1; p62, protein p62; BNIP3, Bcl-2 interacting Protein 3; FUNDC1, FUN 14 domain containing 1; mTOR, Mammalian target of rapamycin; NIX, NIP3-like protein X; HIF-1α, Hypoxia inducible factor-1α; PGC-1α, Peroxisome proliIerators-activated receptor-γ coactivator-α; Mfn, Mitofusin; DRP1, Dynamin-related protein 1; OPA1, Optic atrophy 1; ECM, Extracellular matrix; TGF-β, Transforming growth factor-β; α-SMA, α-smooth muscle actin.

Mitophagy and the NLRP3 inflammasome play crucial roles in maintaining renal homeostasis and responding to renal injury. Mitophagy eliminates damaged mitochondria and reduces ROS production, thereby inhibiting NLRP3 inflammasome activation. This process decreases inflammatory cytokine release and suppresses pyroptosis, ultimately alleviating renal injury and fibrosis (Li et al., 2019; Tian et al., 2023) (Figure 1). The interaction between these two pathways is essential in renal disease pathogenesis. Further investigation of their molecular mechanisms may facilitate the development of novel therapeutic strategies.

### 3.1 The role of mitochondria in renal function

Mitochondria are indispensable organelles in renal cells, critical for physiological kidney functions such as active solute transport and fluid balance regulation (Wang et al., 2024). Furthermore, the kidneys exhibit high energy demands, particularly because the proximal tubules reabsorb the majority of filtered fluid. This process requires substantial oxygen consumption, consequently leading to high mitochondrial density in these regions (Bhargava and Schnellmann, 2017).

Mitochondrial dysfunction is a key factor in the development of AKI (Piret and Mallipattu, 2023), primarily exacerbating renal damage by disrupting energy metabolism and inducing oxidative stress. Mitochondria play a central role in adenosine triphosphate (ATP) synthesis and energy regulation, but dysfunction leads to reduced intracellular ATP production, increased ROS generation, and cellular apoptosis (Aparicio-Trejo et al., 2018). During AKI, the energy demand of renal tubular epithelial cells surges, triggering a metabolic shift from fatty acid oxidation to glycolysis. This shift further impairs mitochondrial function (Chang et al., 2024). Such dysfunction stems from various etiologies (e.g., ischemia or toxin exposure), causing intracellular calcium overload. Calcium overload subsequently activates detrimental enzymes (e.g., phosphatases), compromises cell membrane integrity, and ultimately leads to tubular damage and tissue necrosis (Szydlowska and Tymianski, 2010). Lan et al. (2016) demonstrated that renal IRI induces tubular atrophy accompanied by significant mitochondrial alterations and substantial protein loss. Furthermore, studies confirm that tubular atrophy is closely associated with the metabolic shift from oxidative phosphorylation to glycolysis in renal tubular epithelial cells.

Mitochondrial structural abnormalities play a significant role in the pathological progression of AKI. Healthy mitochondria in the kidneys exhibit high plasticity (McBride et al., 2006), but structural disorders impair ATP synthesis efficiency. Specifically, in AKI models, impaired mitochondrial membrane potential causes matrix swelling and cristae structure disruption. Persistent dysregulation of structural dynamics (e.g., imbalance in mitochondrial fusion/fission) increases ROS generation, which induces renal microvascular loss, oxidative stress, and elevated cell death, ultimately leading to renal failure (Zhang et al., 2021). These structural defects also promote aberrant interactions with other organelles (such as the endoplasmic reticulum), further exacerbating mitochondrial stress and triggering chronic fibrosis during AKI recovery (Zhang et al., 2021).

Mitophagy functions as a selective autophagic process that maintains cellular homeostasis by eliminating damaged mitochondria and exerts a protective role in AKI pathology. Its regulatory mechanisms are intricate and closely linked to disease progression. This process is primarily executed through the PINK1-Parkin pathway and receptor-mediated routes such as BNIP3/NIX. Upon loss of mitochondrial membrane potential, PINK1 accumulates and recruits Parkin to catalyze ubiquitin tagging, followed by LC3 binding to facilitate autophagosome formation (Zhu Q. et al., 2023). During AKI, upregulation of autophagy alleviates oxidative stress and renal fibrosis, as exemplified by α-Klotho, which safeguards renal cells and delays the AKI-to-CKD transition by enhancing autophagic flux. Nevertheless, impaired autophagy after ischemic AKI elevates the risk of post-AKI CKD owing to the ineffective clearance of aberrant mitochondria, resulting in cumulative cellular damage and progressive renal fibrosis (Jiang et al., 2020).

### 3.2 NLRP3 inflammasome

The NLRP3 inflammasome is a multiprotein complex composed of NLRP3, apoptosis-associated speck-like protein (ASC), and pro-caspase-1, and can be activated by the innate immune system to trigger widespread inflammatory responses (Ding et al., 2021). Under physiological conditions, the NLRP3 inflammasome remains inactive. Its stability is maintained through molecular chaperones including heat shock protein 70 and caspase recruitment domain-containing protein 8. This homeostatic regulation prevents excessive inflammation while preserving normal renal function (Kim et al., 2024).

The first priming signal occurs when innate immune cells rapidly detect PAMPs (such as lipopolysaccharide, single-stranded RNA, and bacterial DNA) or DAMPs (including histones, DNA fragments, and heat-shock proteins) via pattern-recognition receptors (primarily Toll-like receptors). This detection activates the NF-κB signaling pathway (Anders and Schaefer, 2014; Li and Wu, 2021; Meng et al., 2025), leading to the upregulation of NLRP3, caspase-1, and pro-IL-1β expression. Concurrently, post-translational modifications such as ubiquitination and phosphorylation maintain NLRP3 in a signaling-competent state (Swanson et al., 2019). Studies indicate that LPS-induced p21-activated kinase 1 phosphorylates caspase-1 at Ser376, leading to downstream NLRP3 activation (Henedak et al., 2024). The second activation signal originates from specific NLRP3 agonists, which trigger NLRP3 inflammasome assembly and downstream activation. This process converts pro-caspase-1 into active caspase-1, which subsequently cleaves pro-IL-1β and pro-IL-18 to generate mature inflammatory cytokines. Notably, activated caspase-1 also cleaves gasdermin D (GSDMD). The N-terminal fragment of GSDMD forms pores in the cell membrane, inducing pyroptosis (He et al., 2015; Gupta et al., 2025).

This cascade of reactions not only enhances host defense by releasing intracellular pathogens and inflammatory mediators (such as IL-1β and DAMPs), but also participates in renal tissue surveillance of DAMPs and PAMPs under normal physiological conditions. It facilitates the clearance of damaged cells and pathogens while promoting tissue repair (Kelley et al., 2019). Under pathological conditions, however, chronic exposure to DAMPs and PAMPs predisposes the NLRP3 inflammasome to activation. This exacerbates inflammatory responses and drives fibrotic progression in kidney disease (Kim et al., 2019).

### 3.3 Interplay between the NLRP3 inflammasome and mitophagy

**FIGURE 2:**
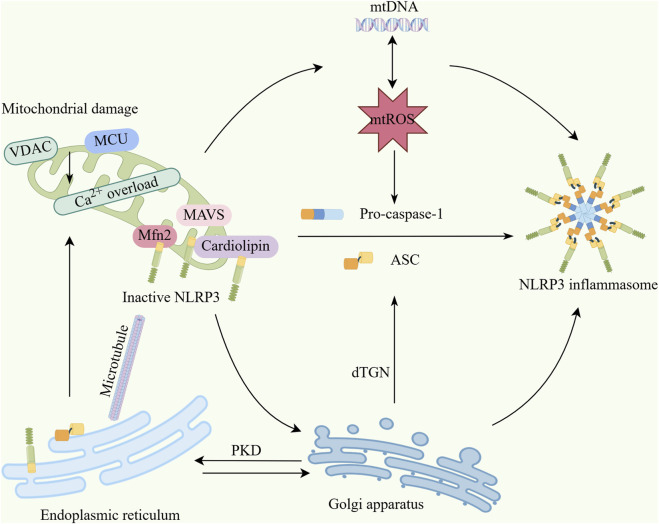
Interaction mechanisms between mitochondria and the NLRP3 inflammasome. (1) Upon mitochondrial damage, factors including VDAC and MCU induce mitochondrial calcium overload, triggering excessive mtDNA release and overproduction of mtROS. These excess mtDNA and mtROS collectively activate the NLRP3 inflammasome. (2) Mitochondria serve as physical platforms for NLRP3 activation, where MAVS, cardiolipin, and Mfn2 bind inactive NLRP3 and recruit it to mitochondria. Damaged mitochondria translocate to the endoplasmic reticulum via microtubules, causing Ca^2+^ overload and decreased mitochondrial stability, which further enhances mtROS production. (3) NLRP3 translocates to mitochondria before dissociating and trafficking to the Golgi apparatus. The trans-Golgi network (TGN) disassembles into dispersed structures (dTGN) upon stimulation. NLRP3 is recruited to dTGN by binding phosphatidylinositol-4-phosphate to form speckles, thereby inducing ASC oligomerization and NLRP3 inflammasome activation. Concurrently, the Golgi recruits mitochondria-associated ER membranes (MAMs) through PKD signaling, also promoting NLRP3 inflammasome activation (By Figdraw). VDAC,Voltage-dependent anion channel; MCU, mitochondrial calcium uniporter; mtROS, Mitochondrial reactive oxygen species; mtDNA, Mitochondrial DNA; MAVS, Mitochondrial antiviral signaling protein; Mfn2, mitofusin-2; ASC, Apoptosis-associated speck-like protein; dTGN, dispered trans-Golgi network; PKD, Protein Kinase D.

The NLRP3 inflammasome can regulate mitophagy to balance necessary host-defensive inflammatory responses and prevent excessive detrimental inflammation. Mitochondrial damage can activate the NLRP3 inflammasome via mtROS. Specifically, mitochondrial antiviral signaling protein (MAVS) or mitofusin 2 (Mfn2) have been shown to recruit NLRP3 to mitochondria during viral infection or NLRP3 stimulation. These MAVS aggregates may facilitate NLRP3 oligomerization to assemble the inflammasome complex (Yu and Lee, 2016). Although ROS generation—particularly mtROS—represents the most well-defined mechanism for NLRP3 inflammasome activation, regulation of the NLRP3 inflammasome by mitophagy extends beyond controlling mtROS production to include modulation of calcium signaling, mtDNA release, and subcellular localization changes (Figure 2).

Mitochondrial damage regulates NLRP3 activity through inhibition of the voltage-dependent anion channel (VDAC) (Zhou et al., 2011). Additionally, autophagy limits inflammasome assembly by degrading NLRP3 and ASC inflammasome components (Cao et al., 2019). Mitochondrial Ca^2+^ uptake is a critical step for NLRP3 activation. Mitochondrial calcium overload induces mitochondrial damage, thereby promoting NLRP3 inflammasome assembly and activation. Concurrently, damaged mitochondria release mtDNA, which can directly bind to and activate the NLRP3 inflammasome. mtDNA release is closely linked to mitophagy deficiency, indicating that mitophagy suppresses NLRP3 activation by clearing damaged mitochondria containing mtDNA (Lawlor and Vince, 2014). Triantafilou et al. (2013) demonstrated that complement membrane attack complex-induced NLRP3 inflammasome activation requires the mitochondrial calcium uniporter (MCU). MCU is essential for mitochondrial Ca^2+^ uptake, and its excessive activation leads to mitochondrial dysfunction. Recent studies reveal that STAT3 protein plays a key role in NLRP3 translocation to mitochondria. STAT3 deficiency inhibits mitochondrial localization of NLRP3, consequently attenuating inflammasome activation. This mechanism underscores the importance of mitochondria as a physical platform for NLRP3 activation (Luo et al., 2024).

During activation, NLRP3 undergoes dynamic subcellular relocalization: it is first recruited to mitochondria, subsequently dissociates, and translocates to the Golgi apparatus. This process likely involves regulation by mitochondrially derived signaling molecules (e.g., cardiolipin) (Zhao and Zhao, 2020; Luo et al., 2024).

The trans-Golgi network (TGN) disassembles upon stimulation, forming dispersed structures (dispersed TGN, dTGN). Subsequently, NLRP3 is recruited to the dTGN and forms speckle structures through binding of its conserved basic amino acid-rich region to negatively charged phosphatidylinositol-4-phosphate. This process induces ASC oligomerization, ultimately leading to NLRP3 inflammasome activation (Chen and Chen, 2018). Concurrently, the Golgi apparatus recruits mitochondria-associated ER membranes toward itself via PKD signaling, establishing connectivity among the “Golgi-MAM-mitochondria” triad, thereby promoting NLRP3 inflammasome activation (Zhang et al., 2017).

Mitophagy deficiency induces cellular metabolic disturbances such as lipid accumulation, which indirectly activates NLRP3 by altering cellular metabolic states including the ATP/ADP ratio (Leishman et al., 2024).

Furthermore, upon stimulation, mitochondria migrate toward the endoplasmic reticulum through the microtubule network. This process induces Ca^2+^ overload and decreased mitochondrial stability, consequently increasing the production of mtROS (Li et al., 2022).

Separate research demonstrates that upon NLRP3 inflammasome activation, mitochondria-associated endoplasmic reticulum membranes become positioned adjacent to Golgi membranes (Zhang et al., 2017). Furthermore, in the resting state, NLRP3 localizes to endoplasmic reticulum structures, whereas upon activation it redistributes—along with the adaptor protein ASC—to perinuclear regions where it co-localizes with clustered endoplasmic reticulum and mitochondria. Mitochondrial dysfunction triggers endoplasmic reticulum stress, thereby exacerbating NLRP3 activation (Elliott and Sutterwala, 2015).

## 4 The Mitophagy-NLRP3 inflammasome axis in AKI-CKD transition

### 4.1 Research advances on the mitophagy-NLRP3 inflammasome axis in diverse AKI-CKD models

In kidney diseases, the mitophagy-NLRP3 inflammasome axis participates in tissue injury, inflammation, and fibrosis processes. Rational modulation of this axis can ameliorate and delay the progression from various AKI models to CKD. Using AKI models induced by IRI, cisplatin, and sepsis-associated (SA) injury as examples, the following section elucidates the role of the mitophagy-NLRP3 axis in renal pathology.

#### 4.1.1 Renal IRI model

The ischemic AKI model is a common system for studying the AKI-to-CKD transition. In this model, ischemia triggers mitochondrial respiratory suppression, causing a sharp decline in ATP production. Concurrently, Na^+^-K^+^-ATPase inactivation induces cellular edema (sodium accumulation reaching 3–4 times normal levels) and structural damage (e.g., brush border loss and podocyte detachment). The S3 segment of proximal tubules is highly susceptible to ischemic necrosis due to limited blood supply (Szeto, 2017). Although reperfusion restores blood flow, activation of the mitochondrial permeability transition pore permits massive Ca^2+^ influx. This initiates oxidative stress (characterized by excessive reactive oxygen species generation) and activates the NLRP3 inflammasome, exacerbating cellular injury (Szeto, 2017; Lv et al., 2021), thereby promoting AKI-CKD progression. Research by Bakker et al. (2014) demonstrates that post-renal IRI, necrotic tissues release endogenous DAMPs such as HMGB1 or mtDNA, which activate the NLRP3 inflammasome in immune cells to mediate renal tubular epithelial cell apoptosis.

Autophagy induced during renal ischemia-reperfusion contributes to protective homeostatic mechanisms under ischemic/hypoxic stress and clears oxidatively damaged proteins and organelles during reperfusion (Ramesh et al., 2019). Following ischemic renal injury, an imbalance in mitochondrial dynamics occurs. Specifically, dynamin-related protein 1 (Drp1)-mediated mitochondrial fission becomes hyperactive, promoting apoptosis and inflammation. Conversely, dysfunction of fusion proteins (Mfn1/2, OPA1) impairs mitophagy and suppresses mitochondrial Ca^2+^ uptake, thereby exacerbating tubulointerstitial inflammation and fibrotic progression (Bhatia et al., 2020). In contrast, Drp1 knockout in renal tubular epithelial cells enhances epithelial repair. This mitochondrial genetic modification concurrently activates the renoprotective β-hydroxybutyrate signaling pathway, mitigating progressive renal injury and fibrosis (Perry et al., 2018). Both *in vivo* and *in vitro* studies demonstrate that NLRP3 inflammasome knockout (Bakker et al., 2014; Zheng Z. et al., 2021) and mitochondrial protection strategies (Szeto et al., 2017) effectively prevent tubular sclerosis and interstitial fibrosis progression post-ischemic injury.

#### 4.1.2 SA-AKI model

Multiple sepsis-associated animal models demonstrate that the mitophagy-NLRP3 inflammasome axis is closely associated with renal inflammation, tubular injury, and dysfunction. Mitochondria constitute the primary source of ROS during sepsis. Both mtROS and mtDNA released upon mitochondrial damage promote NLRP3 inflammasome activation, thereby triggering pyroptosis and exacerbating AKI (Wu W. et al., 2025). In LPS-induced sepsis-associated AKI (SA-AKI) models, DRP1 overexpression causes excessive mitochondrial fission, which releases mtDNA to activate the NLRP3-caspase-1 signaling pathway and ultimately induces pyroptosis-mediated AKI (Liu et al., 2020). Consequently, modulating the mitochondrial quality control system to reduce mtROS and mtDNA release, thus inhibiting NLRP3 activation, may represent an effective therapeutic strategy for preventing SA-AKI progression.

Xu et al. (2024) discovered that tubular-specific TIMP2 knockout mice exhibit more severe renal injury than wild-type mice during the early stage of SA-AKI, accompanied by elevated levels of pyroptotic markers NLRP3, Caspase-1, and GSDMD. Exogenous TIMP2 increases intracellular cyclic AMP (cAMP), promoting ubiquitination of NLRP3 and its autophagy-dependent degradation, thereby attenuating renal tubular pyroptosis and alleviating kidney injury. Notably, oxidative stress—particularly excessive ROS production—plays a critical role in SA-AKI development (Ow et al., 2021).

#### 4.1.3 Cisplatin-induced AKI and unilateral ureteral obstruction (UUO) models

Cisplatin causes dose-dependent nephrotoxicity, and this agent is primarily used to model toxin-induced AKI and its transition to CKD. Cisplatin directly damages renal tubular mitochondria, leading to defective fatty acid oxidation and bioenergetic failure (Chang et al., 2024). In cisplatin-induced mouse models (Li et al., 2019; Ma et al., 2024), proximal tubular cells exhibit ferroptosis, which is closely associated with mitochondrial dysfunction. These mitochondrial abnormalities manifest as reduced size and outer membrane rupture, thereby triggering NLRP3 inflammasome activation and ultimately exacerbating renal fibrosis. In this model, blocking NLRP3 inflammasome activation reduces cisplatin-induced oxidative stress and inflammatory responses, consequently attenuating interstitial fibrosis (Li et al., 2019).

Studies reveal that under UUO model conditions and *in vitro* oxidative stress, mitochondrial damage becomes aggravated (manifested as swelling, cristae fragmentation, and vacuolization). These DAMPs activate the NLRP3 inflammasome (Li et al., 2023). Concurrently, shortening of the 3′UTR region in NLRP3 mRNA increases NLRP3 protein expression, thereby promoting renal injury progression and AKI-CKD transition (Zheng T. et al., 2021). Conditional deletion of autophagy-related proteins 5 and 7 in proximal tubules accelerates renal fibrosis progression in the UUO model, whereas administration of the autophagy inducer rapamycin retards its induced renal fibrotic process (Ramesh et al., 2019).

### 4.2 Mitochondrial autophagy-NLRP3 inflammasome axis-related pathways

The interplay between mitophagy and NLRP3 inflammasome in AKI-to-CKD transition involves both classical pathways (PINK1-Parkin and BNIP3/NIX) and regulatory factors, including Hypoxia-Inducible Factor 1-alpha (HIF-1α) (He et al., 2017), Peroxisome Proliferator-Activated Receptor Gamma Coactivator 1-alpha (PGC-1α) (Lynch et al., 2018), and Mechanistic Target of Rapamycin (mTOR) (Wang et al., 2020). These modulators regulate the mitophagy-NLRP3 axis, influencing renal injury progression or repair (Figure 1).

#### 4.2.1 PINK1/PGC-1α

PINK1 is a mitochondrial kinase that identifies damaged mitochondria and initiates mitophagy. This process is impaired when NLRP3 activation promotes caspase-mediated Parkin cleavage (Liu et al., 2019). In renal proximal tubule cells, Pink1/Parkin knockout causes mitochondrial dysfunction and cellular damage. Conversely, Pink1/Parkin overexpression protects against sepsis-induced mitochondrial and cellular injury (Wang et al., 2021).

Both *in vitro* and *in vivo* studies demonstrate that PINK1/Parkin-mediated mitophagy decreases mtROS and subsequent NLRP3 inflammasome activation (Lin et al., 2019; Zhu D. et al., 2023). This mechanism attenuates apoptosis and tissue damage in cisplatin-induced and ischemia-reperfusion injury models. Parkin silencing or pharmacological inhibition of mitophagy can abolish drug-induced NLRP3 suppression. Such interventions exacerbate NLRP3/caspase-1-dependent pyroptosis (Cheng et al., 2024). Interestingly, PINK1 deficiency may protect against cisplatin nephrotoxicity by preventing excessive mitophagy and caspase-3-mediated apoptosis (Zhou et al., 2019). These divergent effects highlight the context-dependent nature of PINK1/Parkin signaling.

During AKI recovery, damaged mitochondrial clearance and new mitochondrial generation (biogenesis) are essential for renal repair, a process chiefly regulated by PGC-1α. In ischemic and septic AKI recovery, PGC-1α expression gradually normalizes, indicating its protective role in preventing AKI-to-CKD transition (Emma et al., 2016). Through the ERRα-SIRT3 pathway, PGC-1α indirectly stimulates the PINK1-Parkin pathway, enhancing damaged mitochondrial degradation (Chen et al., 2022). PGC-1α maintains mitochondrial quality control by activating mitophagy while suppressing NLRP3 inflammasome activation to attenuate inflammation. In unilateral ureteral obstruction models, PGC-1α overexpression ameliorates TGF-β1-induced mitochondrial dysfunction in renal tubular cells. This improvement is evidenced by restored mitochondrial membrane potential, enhanced oxygen consumption rate, and reduced mtDNA release, collectively inhibiting NLRP3 inflammasome activation and alleviating renal fibrosis (Nam et al., 2022).

#### 4.2.2 BNIP3/HIF-1α

BNIP3 has recently emerged as a novel regulator of mitophagy, though its specific role in renal mitophagy remains poorly understood. Both BNIP3 and FUN14 Domain Containing 1 interact with BCL2 to promote mitophagy. These proteins function by disrupting the Beclin 1-BCL2 interaction, thereby enhancing autophagic activity (Tang et al., 2019). *In vivo* experiments demonstrated that Acyl-CoA Synthetase Family Member 2 (ACSF2) knockout significantly enhanced ischemia-reperfusion (IR)-induced mitophagy, leading to improved renal function in IR-injured mice (Shi et al., 2023). However, when BNIP3 was simultaneously deficient in ACSF2 knockout mice, the IR-triggered mitophagy was suppressed. This dual deficiency ultimately exacerbated renal damage.

In unilateral ureteral obstruction models and hypoxic conditions (Li et al., 2023), BNIP3 gene deletion exacerbates mitochondrial damage, activates the NLRP3 inflammasome, and significantly elevates renal fibrosis markers (α-SMA and TGF-β1). BNIP3 deficiency is strongly linked to programmed cell death pathways. Under non-ischemic conditions, BNIP3 knockout mice showed relatively lower serum creatinine levels compared to sham-operated controls. However, following renal ischemia (Tang et al., 2019), these knockout mice demonstrated significantly higher creatinine levels. They also exhibited increased TUNEL-positive tubular cells and elevated inflammatory factors compared to wild-type mice subjected to ischemia alone.

HIF-1α serves as an upstream regulator of BNIP3, controlling cellular and systemic homeostasis in response to oxygen availability. During hypoxia, HIF-1α expression is upregulated, enabling this transcription factor to bind the hypoxia response element in the BNIP3 promoter region and activate BNIP3 transcription (Liu et al., 2023). HIF-1α overexpression demonstrates complex mechanisms during AKI-to-CKD progression, with model-dependent effects due to variations in hypoxia duration. While some studies report HIF-1α downregulation with elevated IL-1β levels, NLRP3 inflammasome inhibition appears to enhance HIF-1α expression. Current evidence suggests that NLRP3 inflammasome suppression may protect against contrast-induced AKI through upregulation of both HIF-1α and BNIP3-mediated mitophagy (Lin et al., 2021).

In severe AKI models, persistent HIF-1α activation strongly correlates with renal fibrotic lesion development. The C-terminal transactivation domain (C-TAD) of HIF-1α activates KLF5 transcription, a key regulator of cell proliferation, differentiation and fibrotic processes, thus driving AKI-to-CKD progression. During severe IRI, FIH-1 overexpression suppresses HIF-1α C-TAD activity. This inhibition effectively blocks AKI-to-CKD transition (Li Z.-L. et al., 2021). The potential for therapeutic HIF-1α modulation to achieve renal protection requires further investigation.

#### 4.2.3 mTOR

mTOR is a key regulatory molecule in autophagy induction, exerting dual modulation on autophagic activity, with complex mechanisms in kidney diseases (Ramesh et al., 2019). TGF-β1 can both induce collagen synthesis and promote autophagy with subsequent collagen degradation. Studies demonstrate that CI-activated TGF-β1 causes excessive autophagy in renal tubular epithelial cells, thereby exacerbating renal injury (Zhou et al., 2018). Furthermore, TGF-β protein triggers mTOR signaling via the PI3K/Akt pathway, enabling mTOR to bidirectionally regulate autophagy (Ramesh et al., 2019).

α-Klotho protein enhances autophagy by inhibiting the AKT/mTOR pathway, subsequently suppressing NLRP3 inflammasome-mediated pyroptosis and protecting renal tubular epithelial cells (Zhu et al., 2021). Dexmedetomidine augments autophagy through the AMPK/mTOR pathway, inhibiting NLRP3 inflammasome activation and alleviating sepsis-associated kidney injury (Yang et al., 2020). 6-Paradol suppresses renal NF-κB mRNA expression and NLRP3 inflammasome pathway activity, while enhancing renal autophagy by upregulating LC3B, AMPK, and SIRT-1 levels, alongside inhibiting mTOR, p-AKT mRNA expression, and phosphorylated p62 levels (El-Maadawy et al., 2022).

Nuclear factor of activated T-cells (NFAT) activation depends on calcineurin (CaN), and calcium signaling dysregulation is a key trigger for NLRP3 activation (Minami, 2014). In cardiovascular diseases, NFAT regulates cardiomyocyte function via the PI3K/Akt/eNOS/NO pathway, which intersects with mitochondrial homeostasis and inflammasome activation (Walther et al., 2014). Following T11TS treatment in glioma-associated endothelial cells, CaN-NFAT pathway activation may initiate the downstream PI3K-AKT pathway, enhancing T-cell survival and anti-glioma defense (Chaudhuri et al., 2015; Chaudhuri et al., 2018).

In IRI models, deficiency of mitochondrial protein FAM3A disrupts PI3K/AKT/NRF2 signaling, impairs mtROS clearance, and induces NLRP3 inflammasome activation leading to tubular pyroptosis (Li et al., 2024). Enhanced mTOR complex 1 (mTORC1) and rapamycin can block renal fibrosis progression (Ramesh et al., 2019). Given the close association between PI3K/Akt and mTOR pathways, their complex regulatory mechanisms raise a pivotal question: Does NFAT modulate kidney diseases through the PI3K/Akt pathway?

In summary, the mitophagy-NLRP3 inflammasome axis interacts during AKI-CKD transition, co-regulating renal injury progression and repair. Pathways including PINK1/PGC-1α, BNIP3/HIF-1α, and mTOR participate in this process with intricate mechanisms. Calcium signaling dysregulation closely associates with the mitochondrial-NLRP3 axis, prompting a critical inquiry: Does CaN-NFAT regulate kidney diseases via the PI3K/Akt pathway?

## 5 Targeted drugs related to AKI-CKD

Based on the analysis of mitophagy and NLRP3 inflammasome regulation during AKI-to-CKD transition in this review, this section summarizes recent advances in pharmaceutical research targeting these two pathways. It encompasses clinical studies targeting both mitochondria and NLRP3 (Table 1), along with a compilation of relevant models (Table 2).

**TABLE 1:** Clinical trials of agents targeting mitochondria and the NLRP3 inflammasome in kidney diseases.

Inhibitor	Inclusion criteria	Purpose	Mechanism of action	Reference/Clinicaltrial.gov
Elamipretide (SS-31)	14 Patients with ARAS (identified by renal artery Doppler ultrasound velocity acceleration with a mean peak systolic velocity >375 cm/s)	IIa Phase, randomized, double-blind, placebo-controlled clinical trial to evaluate the efficacy of SS-31, in conjunction with PTRA, in improving renal function, oxygenation, and renal blood flow among patients with ARAS undergoing PTRA.	Elamipretide binds to cardiolipin, stabilises it and prevents its peroxidation, stable mitochondrial function	NCT01755858 (Saad et al., 2017)
ASP1128 (MA-0217)	Adult patients with non-emergency coronary artery bypass grafting and/or valve surgery within 4 weeks who have at least one risk factor for AKI (age ≥70 years, eGFR <60 mL/min/1.73 m², congestive heart failure, diabetes mellitus, and/or proteinuria)	A Phase II randomised, double-blind, placebo-controlled, biomarker-driven, multicentre study to investigate the safety and tolerability of ASP1128 in post-cardiac surgery treatment and the pharmacokinetic characteristics of patients at risk of AKI after surgery	Selective PPARδ modulator, promotes fatty acid oxidation	NCT03941483 (van Till et al., 2023)
Nicotinamide	138 adults aged 18 years or older	A Phase II, randomized, double-blind, placebo-controlled clinical trial to evaluate the renal protective efficacy of BASIS™ (nicotinamide riboside and pterostilbene) in patients undergoing complex aortic aneurysm repair and aortic arch reconstruction	Nicotinamide alleviates renal injury caused by oxidative stress by increasing NAD⁺ levels and activating NAD⁺-dependent deacetylases such as SIRT1	NCT04342975 (Pickkers et al., 2022)
Zoledronic acid	68 adult patients with AKI.	A prospective, randomized, double-blind, placebo-controlled trial to evaluate whether zoledronic acid has a renal function–improving effect	A calcium sensitizer that improves renal blood flow and glomerular filtration rate, enhances the activity of mitochondrial respiratory enzymes, reduces mitochondrial free Ca²⁺ levels	NCT01720030
MitoQ	18 patients with CKD (eGFR: 45 ± 3 mL/min/1.73 m²)/	The Effects of MitoQ on Vascular Function and Exercise Capacity in CKD	Mitochondria-derived ROS are reduced, and the activity of NADPH oxidase is indirectly inhibited, concomitant with enhanced mitochondrial uptake	NCT02364648 (Kirkman et al., 2023)
Resveratrol	20 non-dialysis CKD patients (age 62.0 ± 8.0 years, body mass index 27.7 ± 1.2 kg/m², eGFR 34.0 ± 13.0 mL/min)	A randomized, double-blind, crossover trial to assess the effects of resveratrol supplementation on inflammatory and oxidative stress markers in non-dialysis CKD patients	Resveratrol exerts its effects by activating Sirtuin proteins and modulating mitochondrial dynamics	NCT02433925 (Chang et al., 2015)
Verapamil	Patients aged 40 years or older who have hypertension and a known history of type 2 diabetes mellitus for no more than 25 years	A multicenter, double-blind, placebo-controlled, randomized study, it was designed to assess whether ACEI and calcium-channel blockers, used alone or in combination, could prevent microalbuminuria in hypertensive patients with type 2 diabetes who had normal urinary albumin excretion	Prevent the development of microalbuminuria	NCT00503152 (Ruggenenti et al., 2004)

CKD, chronic kidney disease; eGFR, estimated glomerular filtration rate; ROS, reactive oxygen species; NADPH, nicotinamide adenine dinucleotide phosphate; PTRA, percutaneous transluminal renal angioplasty; ARAS, atherosclerotic renal artery stenosis; AKI, acute kidney injury; SIRT1, Silent Information Regulator T1; ACEI, Angiotensin-Converting Enzyme Inhibitor.

**TABLE 2:** Application of drugs targeting mitochondria and the NLRP3 inflammasome in disease models.

No.	Inhibitor	Models	Mechanism of action	References
1	Elamipretide (SS-31)	IRI	SS-31 improves mitochondrial structure and respiratory function, thereby accelerating ATP recovery	Szeto et al. (2011)
2	ASP1128	IRI	ASP1128 increases the expression of genes associated with mitochondrial function	Bracken et al. (2017)
3	Nicotinamide	cisplatin-induced AKI and IRI	Nicotinamide stimulates *de novo* NAD + synthesis by inhibiting ACMSD, thereby improving mitochondrial function	Faivre et al. (2021)
4	MCC950 series	Diabetic Nephropathy、IRI	MCC950 ameliorates renal fibrosis by inhibiting the NLRP3/caspase-1/IL-1β pathway	Zhang et al. (2019), Su et al. (2021)
5	Hederasaponin C	Septic AKI	Hederasaponin C inhibits the expression of TLR4, as well as the activation of the NF-κB and PIP2 signaling pathways, and also suppresses the activation of the NLRP3 inflammasome	Han et al. (2023)
6	Hydroxychloroquine	IRI	Hydroxychloroquine alleviates renal IRI by inhibiting cathepsin-mediated NLRP3 inflammasome activation	Tang et al. (2018a)
7	Dapansutrile (OLT1177)	Folic Acid-Induced AKI-CKD Transition	Dapansutrile targets the NLRP3 inflammasome/caspase-1/IL-1β axis, reduces macrophage infiltration, and modulates autophagy	Elsayed et al. (2021)
8	MitoQ	DN、IRI、cisplatin-induced AKI	MitoQ inhibits the generation of mitochondrial reactive oxygen species (mtROS) and reduces the expression of NLRP3, IL-1β, and TGF-β	Mukhopadhyay et al. (2012), Dare et al. (2015), Huang et al. (2023a)
9	Andrographolide	DN	Andrographolide stabilizes mitochondrial membrane potential, suppresses Fis1, upregulates Mfn2 expression	Liu et al. (2021)
10	Resveratrol	IRI	Resveratrol activates the SIRT1/PGC-1α axis, enhances mitophagy, and suppresses the NLRP3 inflammasome	Chang et al. (2015)
11	Verapamil	IRI	Verapamil enhances mitophagy, reduces intracellular Ca²⁺, inhibits the NLRP3 inflammasome	Song et al. (2021)

IRI, Ischemia-Reperfusion Injury; NLRP3, NACHT, LRR, and PYD, domains-containing protein 3; ACMSD, α-amino-β-carboxymuconate-ε-semialdehyde decarboxylase; NAD⁺, nicotinamide adenine dinucleotide; ATP, adenosine triphosphate; IL-1β, Interleukin-1, beta; LPS, lipopolysaccharide; AKI, acute kidney injury; TLR4, Toll-like receptor 4; NF-κB, Nuclear Factor kappa-light-chain-enhancer of activated B cells; PIP2, Phosphatidylinositol 4,5-bisphosphate; AKI-CKD, acute kidney injury to chronic kidney disease; DN, diabetic nephropathy; mtROS, mitochondrial reactive oxygen species; SIRT1, Sirtuin 1; PGC-1α, Peroxisome proliferator-activated receptor gamma coactivator 1-alpha.

### 5.1 Mitochondria-targeted drugs for kidney diseases

Phosphatidylserine is a phospholipid located in the inner mitochondrial membrane that shows high susceptibility to oxidative damage. When oxidized, it disrupts the phosphatidylserine microdomain on the IMM, resulting in loss of cristae curvature and impaired ETC function (Szeto, 2017). SS-31 (Elamipretide) is a mitochondria-targeted tetrapeptide that enhances ETC efficiency and restores cellular bioenergetics. It specifically binds to phosphatidylserine, preventing its peroxidation and cytochrome c release while preserving cristae structural integrity (Saad et al., 2017). This dual action reduces ROS production while improving electron transport efficiency. In rat models of renal IRI, the mitochondria-targeting peptide SS-31 exerts significant protective effects during early reperfusion. This agent effectively reduces tubular cell apoptosis and necrosis, thereby preventing tubular dysfunction (Szeto et al., 2011). Clinical studies in atherosclerotic renal artery stenosis patients show elamipretide’s therapeutic potential. Compared to placebo, it significantly minimizes ischemic injury, increases estimated glomerular filtration rate (eGFR), reduces renal hypoxia, and improves overall renal function (Saad et al., 2017).

PGC-1α is an important physiological transcriptional regulator of mitochondrial biogenesis. PPARδ regulation has been shown to increase mitochondrial-related gene expression by enhancing fatty acid oxidation, as well as reducing inflammation and fibrosis (Kleiner et al., 2009). ASP1128 is an effective selective PPARδ modulator. Non-clinical pharmacology data generated by ASP1128 indicate (Bracken et al., 2017; Li Y. et al., 2021) that selective PPARδ modulation following ischemic AKI events in rats can restore renal tubular function, increase the expression of PPARδ target genes (including mitochondrial function-related genes) in blood and kidney tissues, and improve renal tissue pathology. However, in a trial involving patients at risk of AKI following cardiac surgery (van Till et al., 2023), although the incidence of atrial fibrillation was lower in the ASP1128 group, the incidence of adverse renal events in the ASP1128 group was 13%, which was higher than the 11% incidence in the placebo group. This study demonstrated that ASP1128 is safe and well-tolerated in patients; however, further investigation is needed regarding renal adverse events.

Nicotinamide adenine dinucleotide (NAD^+^) is a mitochondrial coenzyme that participates in electron transport and serves as a substrate for deacetylases and poly (ADP-ribose) polymerases (PARPs). It plays essential roles in regulating mitochondrial biogenesis, metabolism, and energy production (Faivre et al., 2021). NAD phosphate (NADP^+^) is vital for maintaining detoxification and antioxidant systems. To fulfill these functions, cells maintain NADP^+^ primarily in its reduced form (Fontecha-Barriuso et al., 2021). Alterations in NAD^+^ synthesis serve as biomarkers for both AKI and CKD models, with particularly low levels observed in renal tissue. While nicotinamide demonstrates protective effects in cisplatin and IRI-induced AKI by mitigating mitochondrial damage, it fails to ameliorate UUO-induced CKD progression (Faivre et al., 2021). This limitation may stem from irreversible mitochondrial damage and renal tubular atrophy in CKD models. Nicotinamide supplementation shows therapeutic potential for ischemic AKI, and its application in preventing AKI-to-CKD transition warrants further investigation. Clinical studies have explored NAD^+^ supplementation for preventing AKI following aortic aneurysm repair surgery (Hariri and Legrand, 2025), highlighting its translational relevance.

Levosimendan is a non-selective ATP-sensitive potassium channel agonist (Pickkers et al., 2022). Experimental studies demonstrate that it attenuates AKI development following cardiac injury in rat models. This protective effect is mediated through multiple mechanisms, including enhancement of mitochondrial respiratory enzyme activity and improvement of mitochondrial energy metabolism. Furthermore, levosimendan regulates mitochondrial dynamics by modulating key protein expression. It decreases Drp1 expression while increasing Opa1 expression (Zhao et al., 2021), thereby promoting mitochondrial stability. A clinical trial (NCT01720030) is currently investigating the potential renal protective effects of dexmedetomidine (Zeximend), although results are pending.

### 5.2 NLRP3 inflammasome-targeted therapeutic strategies and inhibitors in kidney disease

Most NLRP3 inflammasome inhibitors remain in preclinical development. The following section evaluates their therapeutic potential for kidney diseases based on *in vitro* and *in vivo* experimental data. MCC950, a selective NLRP3 inflammasome inhibitor, demonstrates beneficial effects in early-stage diabetic nephropathy (DN) models. It reduces albumin-to-creatinine ratio (ACR) and urinary neutrophil gelatinase-associated lipocalin levels in diabetic mice, while improving renal function and attenuating podocyte injury and fibrosis (Zhang et al., 2019). However, MCC950 shows contrasting effects in established DN models. In these cases, it may aggravate renal inflammation and damage, evidenced by mesangial expansion and worsened glomerulosclerosis (Østergaard et al., 2022). These findings indicate the need for further investigation into its mechanism of action. Additional studies reveal that MCC950 treatment protects against ischemia-reperfusion (I/R) induced renal injury. In renal I/R mouse models, it significantly reduces cytokine release and cellular apoptosis (Su et al., 2021).

Hederasaponin C (HSC) is a natural compound with demonstrated anti-inflammatory and antioxidant properties. In renal injury pathogenesis, TLR4 activation serves as a critical initiating event, subsequently inducing NLRP3 inflammasome assembly through downstream signaling cascades (Han et al., 2023). The NLRP3 inflammasome functions as a central effector in the innate immune system’s renal inflammatory network. Its activation mechanism involves phospholipase C gamma 2 -mediated phosphatidylinositol 4,5-bisphosphate hydrolysis. This process generates second messengers inositol trisphosphate and diacylglycerol, which promote Ca^2+^ release from endoplasmic reticulum stores, ultimately triggering NLRP3 inflammasome activation (Yuan et al., 2022). Research demonstrates that HSC specifically binds to TLR4’s extracellular domain. Through competitive inhibition of TLR4 activation, HSC significantly suppresses NLRP3 inflammasome activity (Han et al., 2023).

Hydroxychloroquine (HCQ), a commonly used antimalarial drug, exhibits potent anti-inflammatory properties. It ameliorates renal IRI by suppressing cathepsin-mediated NLRP3 inflammasome activation (Tang T. T. et al., 2018). As a well-characterized autophagy inhibitor, HCQ administration post-reperfusion or reoxygenation causes abnormal accumulation of microtubule-associated protein 1 light chain 3-II (LC3-II) and sequestosome-1 (p62). This disrupts autophagosome-lysosome fusion, leading to intracellular accumulation of autophagic vesicles and impaired autophagy flux (Tang T. T. et al., 2018). Numerous studies have demonstrated the renal protective effects of autophagy (Jiang et al., 2012; Zhao et al., 2018). However, the therapeutic potential of HCQ in kidney diseases requires further investigation, as its effects may vary depending on ischemic severity, I/R injury stage, and other undefined factors.

Dapansutrile (DAPA) represents the first NLRP3 inflammasome inhibitor to reach Phase II clinical trials (Klück et al., 2020). Its mechanism of action involves inhibiting ATPase activity, thereby interfering with inflammasome component oligomerization. In folate-induced nephropathy models, increased microtubule-associated protein1 LC3-II expression suggests compensatory autophagy activation in response to caspase-1/IL-1β/IL-18 inflammasome pathway stimulation. DAPA treatment significantly reduces LC3-II accumulation (Elsayed et al., 2021), demonstrating its potential to modulate autophagic processes through NLRP3 inflammasome inhibition.

### 5.3 Therapeutic strategies targeting the Mitophagy-NLRP3 inflammasome axis

Mitoquinone (MitoQ) is a targeted antioxidant that specifically accumulates in mitochondria due to its positively charged property. Within the mitochondrial matrix, MitoQ is reduced to its active ubiquinol form by the electron transport chain, effectively preventing oxidative damage through continuous ubiquinone-ubiquinol redox cycling (Chen et al., 2024). Thioredoxin-interacting protein (TXNIP) serves as a key activator of the NLRP3 inflammasome pathway. MitoQ inhibits activation of the mtROS-TXNIP/NLRP3/IL-1β axis, thereby alleviating tubular injury in DN (Han et al., 2018; Huang G. et al., 2023). Studies demonstrate that MitoQ pretreatment significantly mitigates mitochondrial structural and functional damage in renal tubules of mice. This protective effect was observed in both ischemia-reperfusion (Dare et al., 2015) and cisplatin-induced (Mukhopadhyay et al., 2012) kidney injury models, reducing oxidative stress and local inflammation while suppressing NLRP3 inflammasome activation. In a clinical trial involving stage 3–4 CKD patients (Kirkman et al., 2023), MitoQ administration improved macrovasculature endothelial function and arterial hemodynamics. The observed enhancement in microvascular function was partially mediated by reduced NADPH oxidase activity.

Andrographolide is a natural compound extracted from Andrographis paniculata, exhibiting anti-inflammatory and anti-diabetic activities. In both *in vivo* and *in vitro* models of DN, this agent ameliorates mitochondrial dysfunction, stabilizes mitochondrial membrane potential, and eliminates high glucose-induced mitochondrial dynamics abnormalities in HK-2 cells. Specifically, it suppresses expression of mitochondrial fission protein Fis1 while enhancing expression of fusion protein Mfn2. Furthermore, andrographolide reduces ECM accumulation, thereby inhibiting mtROS-mediated NLRP3 inflammasome activation (Liu et al., 2021).

Resveratrol is a natural polyphenolic compound that activates SIRT1 to stimulate PGC-1α activity and mitochondrial biogenesis. This activation enhances mitochondrial fatty acid oxidation capacity in renal cells (Huang J. et al., 2023), while alleviating toxin-induced mitochondrial fission and promoting fusion processes (Zhang et al., 2020). Chang et al. (2015) reported that resveratrol enhances mitophagy through the p38 mitogen-activated protein kinase signaling pathway, promoting clearance of damaged mitochondria and inhibiting NLRP3 inflammasome activation. These effects are reversible by autophagy inhibitors. However, clinical trial data (NCT02433925) (Saldanha et al., 2016) demonstrated no significant improvement in inflammatory or oxidative stress markers in CKD patients receiving resveratrol treatment. Optimization of dosing regimens and treatment duration is required to comprehensively evaluate resveratrol’s therapeutic potential in CKD management.

Mitochondrial calcium overload is a critical factor for NLRP3 inflammasome activation. Ultraviolet radiation and cholesterol-dependent cytolysin-induced NLRP3 activation require intracellular Ca^2+^ influx (Elliott and Sutterwala, 2015). The calcium channel blocker verapamil reduces intracellular Ca^2+^ levels to enhance autophagic flux and suppresses TXNIP expression, thereby inhibiting NLRP3 inflammasome activation. This effect prevents apoptosis in proximal tubular epithelial cells and ultimately ameliorates interstitial fibrosis (Song et al., 2021). In the multicenter, double-blind, randomized Bergamo Nephrologic Diabetes Complications Trial (BENEDICT), patients receiving verapamil combined with trandolapril exhibited nearly 20% lower risk of microalbuminuria compared to trandolapril monotherapy (Ruggenenti et al., 2004).

## 6 Therapeutic potential and challenges

Recent years have witnessed substantial advances in renal disease research, particularly regarding molecular mechanisms and clinical translation. A deeper understanding has emerged of mitophagy and the NLRP3 inflammasome pathway. However, the intricate bidirectional regulatory networks and dynamic equilibrium mechanisms governing these processes remain largely elusive. From a translational perspective, the dual nature of mitophagy - exhibiting both protective and detrimental effects - poses significant challenges. Current NLRP3 inhibitors face limitations due to their inadequate target specificity, creating a substantial translational gap between preclinical findings and clinical applications. Furthermore, existing animal models fail to fully recapitulate the disease heterogeneity and microenvironmental complexity observed in human nephropathies, which impedes comprehensive mechanistic investigations.

Mitophagy is generally regarded as a cytoprotective mechanism (Suman et al., 2024). However, its dual role in regulating cell death has led to conflicting research findings. In a mouse model of bilateral renal ischemia (30 min) followed by 48-h reperfusion, researchers observed increased mitochondrial and autophagic vacuoles in renal tubules, confirming autophagic activation (Tang C. et al., 2018). Conversely, Zeng et al. (2014) demonstrated that autophagy inhibitors ameliorate aristolochic acid-induced cell death during late-stage AKI. These studies indicate that mitophagy’s role in AKI is context-dependent. Its impact on cell survival or death is modulated by multiple factors, including stress severity, cell-type specificity, and expression profiles of autophagy-related molecules. In IRI models, the consequences of autophagy modulation may depend on ischemic duration—prolonged ischemia can trigger autophagy-dependent cell death or cause autophagic impairment associated with post-reperfusion autophagosome accumulation (Ramesh et al., 2019).

Research on NLRP3 inflammasomes also faces similar translational challenges. Although the activation mechanisms of NLRP3 inflammasomes and their role in diseases are relatively well-defined, the development of related inhibitors still faces significant challenges. Animal studies have demonstrated that NLRP3 inhibitors, such as MCC950, exhibit significant therapeutic effects in models of chronic renal failure (Sabra et al., 2023) and diabetic nephropathy (Zhang et al., 2019). However, there is a severe lack of clinical data on their efficacy in human kidney diseases, and these inhibitors have not yet been approved by the U.S. Food and Drug Administration (FDA) or other regulatory agencies (Das et al., 2021). Additionally, NLRP3 inhibitors are prohibitively expensive, which complicates large-scale production and would impose a substantial financial burden on patients in clinical settings (Das et al., 2021). Furthermore, their therapeutic efficacy is highly time-sensitive, creating significant obstacles for clinical application. While the inflammatory response following AKI has been extensively studied, the precise mechanisms by which soluble inflammatory mediators and immune cells drive the progression from AKI to CKD remain unclear (Kurzhagen et al., 2020).

Current animal models for studying the AKI-to-CKD transition have significant limitations. Studies have shown that UUO and bilateral IRI models can effectively induce chronic pathological changes, such as renal fibrosis, but these studies typically assess outcomes only within 6 weeks, lacking long-term follow-up data (Burne-Taney et al., 2005). In contrast, the unilateral IRI model can mimic certain pathological features of AKI-CKD progression, but technical challenges hinder continuous monitoring of dynamic renal function changes (Liang and Liu, 2023; Koh and Chung, 2024).

These limitations hinder our in-depth understanding of the molecular mechanisms driving AKI-to-CKD progression. Additionally, significant variations in experimental conditions between studies, including differences in ischemia duration and temperature control, make direct comparison of research findings challenging. More critically, existing animal models predominantly use healthy young subjects, while clinical AKI patients are typically elderly individuals with multiple comorbidities, such as hypertension and diabetes mellitus (Sánchez Horrillo et al., 2022). This substantial disparity between experimental models and clinical reality may compromise the translational relevance of mechanistic studies.

## 7 Conclusion

This study elucidates the bidirectional regulatory mechanisms between mitophagy and the NLRP3 inflammasome during the progression from AKI to CKD, it also assesses their therapeutic potential. Importantly, clarifying the molecular threshold at which autophagy transitions from a protective to a detrimental effect is crucial for renal repair. Current research predominantly focuses on the regulation of inflammation by mitophagy, while the feedback effects of inflammation on mitochondrial function remain understudied. Further investigation into this interaction mechanism is warranted.
